# Microbial community structure shows differing levels of temporal stability in intertidal beach sands of the grand strand region of South Carolina

**DOI:** 10.1371/journal.pone.0229387

**Published:** 2020-02-27

**Authors:** Harrison B. Taylor, Harry D. Kurtz

**Affiliations:** Department of Biological Sciences, Clemson University, Clemson, SC, United States of America; University of Minnesota Twin Cities, UNITED STATES

## Abstract

Studies of microbial community structure in intertidal and supratidal beach sands along the California and Gulf of Mexico coasts have begun to reveal geographical patterns in microbial diversity through the use of next generation sequencing technology. Only a few studies have targeted communities along the Eastern seaboard, leaving a variety of microbial ecosystems uncharacterized. In this study, we examine the microbial community structure within three South Carolina beaches along the Grand Strand via sequencing of the V4 region of the 16S rRNA gene to discern relationships between diversity and temporal or regional factors. *Gammaproteobacteria*, *Planctomycetes*, *Acidobacteria*, and *Actinobacteria* dominated the composition of these beaches. Diversity analyses revealed that highly diverse communities were similar in overall composition and diversity but showed different levels of community structure stability over time. The community structure in Pawleys Island sands showed no significant change over time, while Garden City experienced significant shifts between each sampling date. Community structure also differed between beaches and, to a lesser degree, sampling date. These data provide evidence of the high microbial diversity within these beach sands and suggest that even though beaches of the same geographic region can show similarity in composition and diversity at a particular timepoint, the nature of their community structure and underlying diversity may differ comparatively and over time.

## Introduction

Marine beaches represent transitional ecosystems that connect terrestrial and marine environments and are widespread in the United States, which has a coastline extending over 95,000 total miles [1]. In addition to ecosystem services and trans-ecosystem nutrient exchange, coastal ecosystems provide support to the economy, generating a GDP in excess of $300 billion annually [2]. Therefore, studying these ecosystems and the community of organisms that comprise them will aid conservation efforts that will help maintain the tourism industry in these areas, as poor ecosystem health can pose a threat to the health of the public, which can impact tourism and recreation. There is evidence that microbial diversity is intrinsically linked to ecosystem function and stress resistance [3,4,5], so proper maintenance and preservation of diverse communities will keep the ecosystem healthy, which will in turn minimize swim advisories and beach closures, benefiting the tourism industry. While there has been extensive research on microbial communities in marine coastal waters [6,7,8,9,10], there is still much to be discovered about microbial communities within the dry and intermittently wet sands bordering these ecosystems. Sandy beach ecosystems represent a transitional link between land and sea, not only providing a means of nutrient transfer between them but also provide important ecosystem services like water filtration and purification and nutrient cycling and mineralization [11,12,13,14]. These processes are carried out by both microbial activity and hydrological processes [11,13]. The hydrology of marine beach ecosystem creates a dynamic topographical structure comprised of distinct areas having unique physical and chemical features, including the supratidal zone, the intertidal zone, and subtidal zone [14,15]. The supratidal zone, located in the backshore, is comprised of a superficial layer of comparatively drier sand overlying the intertidal zone sands, which are subject to sporadic wetting from tidal action. A dense saltwater wedge comprises the majority of the subtidal zone, which intermingles with the overlying freshwater to allow for the exchange and transport of both nutrients and microbes to and from off-shore areas [13,14,16].

Previous studies on beach sands have focused primarily on the presence of indicator organisms or pathogens [15,17,18,19], although studies on whole microbial communities, including members that perform the services that support these ecosystems, are becoming more prevalent [20,21]. Many of the microbial community studies of beach sands have been limited to subtidal sands [12,22,23], but intertidal sands house large and diverse communities as well [13,14,24] and are subject to more human interaction [15]. Collectively, these studies show that beach based microbial communities have high levels of richness and diversity [12,13,14,22,24,25,26] due in part to the introduction of microbes from adjacent ecosystems and their ability to attach and colonize the surface of sand grains [25,27,28]. Beach sand communities are influenced by a number of factors, including geographic location [14,26,29], temporal changes like seasons and temperature [12,22,26], human interaction [26], and disasters like oil spills [24,29] or hurricanes [30]. In the face of such disturbances, a higher level of ecosystem diversity seems to confer a level of resiliency and speed of recovery in the face or large disturbances [5,30].

Diversity within the microbial community of beaches has previously been found to vary over time at higher taxonomic levels (family and genus levels), while tending to maintain relatively stable compositions of lower taxonomic levels like phylum and class [12,22]. Despite this variation, there exists a “functional redundancy” where the ecosystem functions attributed to the microbes present, including nitrogen cycling [13,26] or hydrocarbon degradation [13,24,29], remain constant [22]. The most common phyla comprising these marine beach sands are *Proteobacteria*, *Planctomycetes*, *Bacteroidetes*, *Acidobacteria*, and *Actinobacteria* [13,14,24,26], with the *Gammaproteobacteria* class comprising a large percentage of these communities [24,26,29]. Staley and Sadowsky [14] found sands at two Florida beaches to have higher levels of *Deltaproteobacteria* and *Firmicutes* compared to beaches along the Pacific Ocean or those of the Great Lakes. In their extensive study of 49 California beaches, Boehm *et al*. [13] discovered approximately 1,000 different microbes comprising a cosmopolitan population across all communities.

Staley and Sadowsky [14] found regional differences in bacterial community composition between not only freshwater and marine beaches but also those on different sides of the country. Even within the same beach, communities can be different between the sand and the adjacent seawater [26] and between different depths within the sand [22]. Beach sand communities are reportedly more diverse than communities in the adjacent seawater [24,26]. Reports on differences between communities of different depth profiles remain mixed, with Böer *et al*. [22] reporting different community structures between different depths in subtidal sands and Staley and Sadowsky [14] reporting a minimal role of depth in shaping community structure of sands above the subtidal zone. Other factors influencing these differences of these communities include sand grain size, beach hydrology, physicochemical parameters, and the degree of human interaction [13,14].

This study describes the microbial diversity within marine beach sands from the intertidal and supratidal zones of three South Carolina beaches in the same geographic region. The beaches selected are in a region of the country that has seldom been studied with regard to microbial community structure. Samples were taken at different seasons and at different areas of the beach to investigate if these communities were influenced by temporal or spatial factors. We expect to find a core community within this geographical region as other such core communities have been described in beach systems [13]. We hypothesize that these communities will show differences in overall structure on a seasonal basis, though human and weather related impacts are confounding variables. This study characterizes a set of microbial beach sand communities not previously studied, expanding the map of characterized coastal microbiomes. These data will serve as a baseline on which to rest future studies examining the effects of a variety of impacts, such as, beach restoration efforts, increased tourism and sea level rise.

## Materials and methods

### Sample sites, collection, and processing

Samples were collected from three beaches along the Grand Strand region of South Carolina (Table 1) on four sampling dates: September 10, 2016; January 3, 2017; April 26, 2017; and September 22, 2017. It is important to note that sands from January 3, 2017 were collected about three months after Hurricane Matthew, which had a severe impact on the area, and Garden City sands from September 22, 2017 were collected in the middle of a beach renourishment project, during which several thousand cubic meters of sand from offshore were dumped onto the beach in an effect to alleviate the effects of Hurricane Matthew. These sampling sites were chosen because they are all within close proximity of each other (~32 km radius) and all represent subtropical recreational beaches that experience a moderate to large amounts of tourism. No permits were required for sampling, as the three beaches are public access beaches. The site at Pawleys Island represents a barrier island beach that experiences much less tourism and recreation throughout the year than the other two sites. The island is strictly residential and absent of large infrastructure of commercial buildings. The site at Garden City represents a barrier peninsula beach, and the site at Myrtle Beach represents a mainland beach. The latter two sites experience a greater amount of tourism throughout the year and are more urbanized with more infrastructure than the Pawleys Island site.

**Table 1:** Sample site information.

Location	Beach	Latitude (DMS)	Longitude (DMS)
Myrtle Beach, SC	Springmaid Pier	33° 39’ 35.9994” N	78° 55’ 12” W
Garden City, SC	Garden City Pier	33° 34’ 46.0020” N	78° 59’ 42.3240” W
Pawleys Island, SC	The point at Pawleys Inlet	33° 23’ 59.9994'' N	79° 8' 24” W

Samples were collected during low tide from six different areas of the beach: approximately 10 cm and 50 cm down in the supratidal and high tide zones, and approximately 10 cm deep in the mid-tide and low tide zones. These sampling depths were chosen because they were more likely to represent a more permanent community than those at shallower levels. Samples from each of these six areas of the beach were taken in duplicate approximately 2–3 m apart and pooled together. Tidal zones were located approximately 5 m apart at each beach. Samples were collected using an ethanol sanitized shovel and were stored in zip-lock or Whirl-Pak backs and transported back to the lab on ice. Seawater temperature was measured at each beach on each sampling date at the time of sampling with a Rayteck Raynger® ST^TM^ portable infrared thermometer (Fluke Process Instruments, Cambridge, UK). Once the samples reached the lab, they were processed, with a portion of the sand being stored at -80°C prior to DNA extraction. The remaining sand was measured colorimetrically for the concentration of ammonium, nitrate, and nitrite present in the sand, according to Gerhardt *et al*. [31] and Kartal *et al*. [32].

### DNA sequencing and analysis

Genomic DNA was isolated from 0.50–0.75 g of each sample of sand using the DNeasy® PowerLyzer® PowerSoil® Kit (QIAGEN; Hilden, Germany) according to the manufacturer’s instruction. DNA samples were diluted to 1 ng μl^-1^ and prepared for sequencing using a barcoded 16Sf/16Sr primer set targeting the V4 region of the 16S rRNA subunit according to Kozich *et al*. [33]. Sequencing was performed on a MiSeq V2 2x250bp Illumina platform (San Diego, CA, USA) at Clemson University. Sequence processing and analysis was performed using version 1.35.1 of the mothur software package [34,35]. The SILVA database (version 132) was used as a reference to perform sequencing alignment and classify sequences into operational taxonomic units (OTUs) using a 0.03 cutoff [36]. When needed, sequences were compared to GenBank database using BLAST [37]. Chimera removal was performed through mothur using UCHIME software [38]. Prior to diversity analysis, sequences classified to organisms other than *Bacteria* and *Archaea* (eukaryotes, chloroplast, mitochondria, and unclassified sequences) were removed.

### Statistical and diversity analysis

Alpha (α) and beta (β) diversity analyses and statistics were performed using mothur on a randomized subsample of 6,930 sequences per sample [34,35]. Good’s coverage for each sample after rarefaction ranged from 78.5% to 96.1%. Richness estimates were calculated in mothur using the Chao1 richness estimate. Evenness was evaluated using the Shannon and Inverse Simpson indices [39]. Beta diversity was measured and compared based on Theta-yc (Θ_YC_) distance [40]. Principal coordinate analysis (PCoA) was used to create ordination plots describing the diversity with analysis of molecular variance (AMOVA) used to determine clustering of the samples [41]. The Spearman method was employed to determine which OTU or OTUs most strongly affected the clustering of the different samples. Supplemental analyses of alpha and beta diversity were performed using QIIME 2 2018.8 [42], in which demultiplexing, quality filtering, denoising, alignment, and classification were performed prior to diversity analysis. The number of observed OTUs, Faith’s Phylogenetic Diversity [43], and Shannon indices were estimated at a sampling depth of 2,000 sequences per sample, as this was approximately the number of sequences in the smallest-sized sample within the QIIME2 dataset. Weighted and unweighted Unifrac analyses were used to analyze beta diversity and community structure significance [44]. A canonical correspondence analysis (CDA) was performed based on Bray-Curtis dissimilarity using the BiodiversityR package on RStudio [45,46].

### Accession numbers

Sequence data from this study have been deposited at DDBJ/ENA/GenBank under the following accession numbers: KCJV00000000, KCJX00000000, and KCJW00000000. The versions described in this paper are KCJV01000000, KCJX01000000, and KCJW01000000, respectively. Raw sequence reads used for the analyses can be found under accession numbers PRJNA488273, PRJNA488276, and PRJNA488278.

## Results

Environmental parameters are displayed in S1–S3 Tables. With a few exceptions, ammonium content remained similar across all samples collected for each sampling site. Most of the fluctuation in nitrogen content across seasons occurred in sand samples from the supratidal zone. No clear trends appear in ammonium content with the exception that Myrtle Beach sands tended to have higher levels in September 2016 and Garden City and Pawleys Island tended to have higher levels in sands collected September 2017.

At all beaches, the nitrite content tended to decrease significantly (p < 0.05, One-way ANOVA) from September 2016 to January 2017, with more pronounced decreases occurring above the intertidal zone. Most nitrite levels increased again in warmer months, and at the low and mid-tide zones of all beaches, nitrite content from September 2017 sands exceeded that of sands from the previous September. Nitrite levels also tended to be higher in sands above the intertidal zone than those at the lower end of the intertidal zone (mid- and low tide sands). With the exception of samples from September 2017, Pawleys Island sands tended to have lower nitrite levels. Nitrate tended to be more concentrated in September 2016 and January 2017, with a downward trend in concentration in April and September 2017. Nitrate levels were lowest among all samples in sands collected September 2017.

### Microbial community composition of beach sands

**Fig 1:**
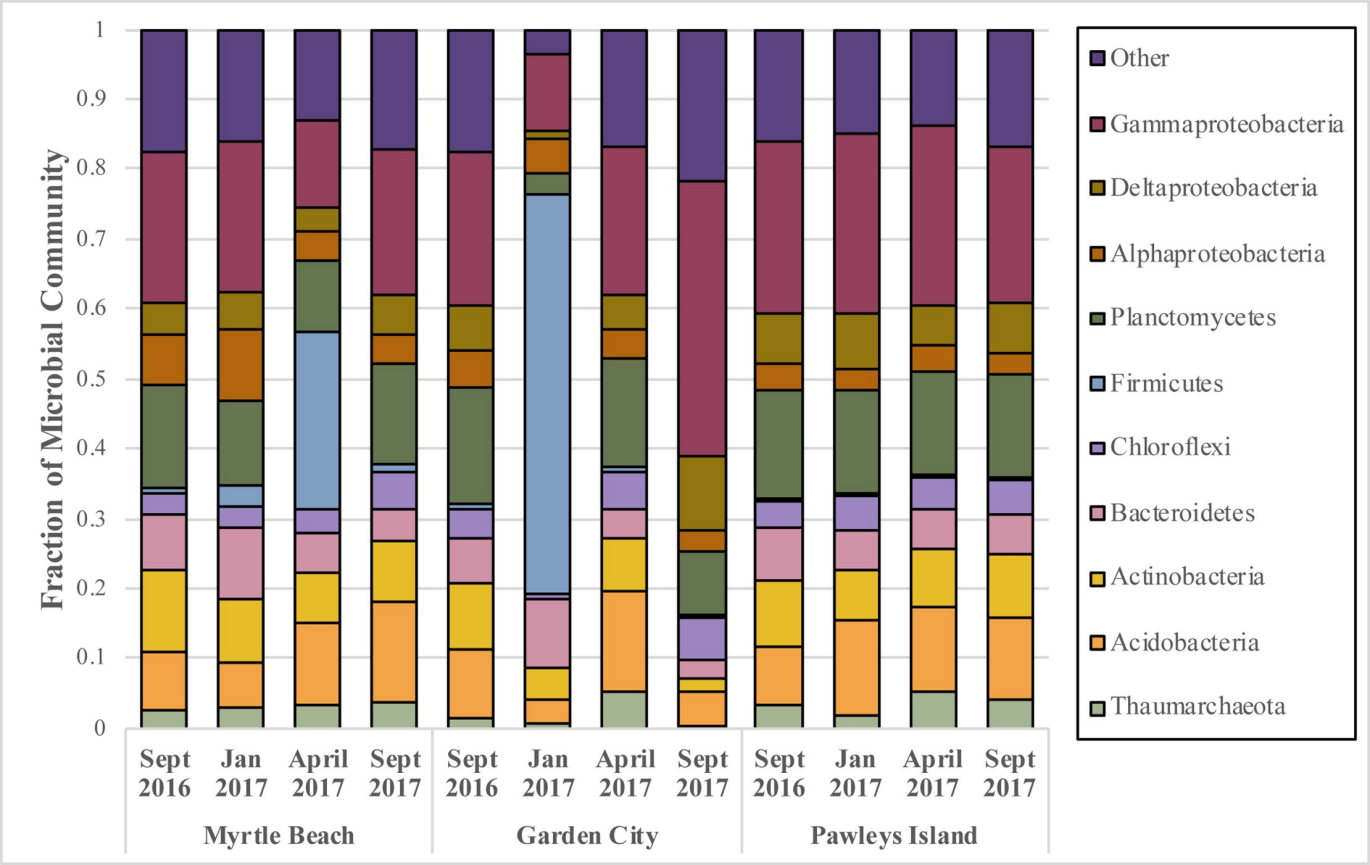
Seasonal phylum composition for all sand samples pooled together from each sampling date. Sampling dates include September 2016, January 2017, April 2017, and September 2017. The *Proteobacteria* phylum has been divided into classes to display members making up ≥ 1% of the community.

Sequencing analysis identified 62 total classified bacterial phyla and 10 total classified archaeal phyla from 3,385,703 total sequences and 78,207 unique OTUs from 71 total beach samples. The most abundant phylum based on percent composition was *Proteobacteria*, which comprised on average over 20% of the bacteria recovered from each beach across all sampling dates (Fig 1). *Gammaproteobacteria* made up the bulk of the proteobacterial population, comprising at least 19% of the total composition at each beach in combined samples (S1 Fig). Unclassified bacterial sequences comprised 3–4% of the total community recovered from each beach. Other phyla making up a large percentage of the community from each beach include *Planctomycetes* (10.2–14.5%), *Acidobacteria* (7.4–11.4%), *Actinobacteria* (6.0–8.5%), and *Bacteroidetes* (5.8–6.2%), and *Chloroflexi* (3.2–4.1%). The most abundant archaeal phyla were *Nanoarchaeaeota*, *Euryarchaeota*, and *Thaumarchaeota*. The *Thaumarchaeota* were the most dominant archaeal phylum present, comprising between 0.82 and 5.2% of the total microbial community of each beach.

Fig 1 also shows the phylum level compositional differences between the beaches. *Firmicutes* showed a large range of variability in its relative abundance at each beach. In Pawleys Island sands, this phylum comprised at most 0.3% of the total community at any sampling date. At Garden City and Myrtle Beach, *Firmicutes* levels are comparable to that of Pawleys Island except in the Myrtle Beach sample from April 2017, where it comprised 25.4% of the population, and in the Garden City sample from January 2017, where it comprised 56.8% of the population. When all sampling dates are combined, *Firmicutes* made up 20.9% and 7.2% of the total communities at Garden City and Myrtle Beach, respectively. The communities at Myrtle Beach and Pawleys Island compensated for the comparative lack of *Firmicutes* with higher compositions of *Acidobacteria*, *Actinobacteria*, *Cyanobacteria*, and *Planctomycetes*. Pawleys Island had an overall higher composition of *Gamma*- and *Deltaproteobacteria* than the other two beaches. Pawleys Island additionally had a higher composition of *Planctomycetes*, while the microbial community at Myrtle Beach had a higher percentage of *Alphaproteobacteria* than the other beaches.

Of the three beaches, Pawleys Island appeared the most stable in composition at all classification levels across all four sampling dates (Fig 1, S2 Fig, S3 Fig). The Myrtle Beach community showed similar compositional make-up to that of Pawleys Island on all sampling dates except on April 2017, where it experienced a large increase in *Bacillaceae* (phylum *Firmicutes*). Garden City appeared the least stable in composition, experiencing large changes in community member composition between all sampling dates, primarily influenced by increases in *Firmicutes* (primarily *Bacillaceae*) on January 2017 and *Alteromonadales* (phylum *Gammaproteobacteria*) on September 2017.

Other similarities and differences in community composition at these taxonomic levels between beaches and sampling dates can be seen in S1–S3 Figs. Aside from the elevated levels of *Bacillales* and *Alteromonadales* in certain samples, *Pirellulales* (1.3–12.8%), *Steroidobacterales* (0.3–7.4%), *Actinomarinales* (0.6–9.8%), and *Thermoanaerobaculales* (0.4–10.6%) were the most abundant orders across all samples in these communities. Overall, Pawleys Island had higher levels of *Steroidobacterales* and *Thermoanaerobaculales* (averages of 5.2 and 6.0%, respectively) compared to Myrtle Beach (3.0 and 3.1%) and Garden City (3.1 and 3.0%). The most consistently abundant taxa classified at the family level include *Pirellulaceae* (1.3–12.8%), *Woeseiaceae* (0.3–7.4%), *Thermoanaerobaculaceae* (0.4–10.6%), and *Nitrosopumilaceae* (0.1–6.9%). Myrtle Beach and Garden City tended to have higher levels of *Flavobacteriaceae* and *Rhodobacteraceae* compared to Pawleys Island, which tended to have higher levels of *Woeseiaceae* (Order *Steroidobacterales*) and *Thermoanaerobaculaceae*. The most abundant OTU was a member of the *Woeseiaceae* family, classified as a *Woeseia* sp., and was commonly detected in all samples, comprising 3.9% of all classified sequences recovered.

### Microbial diversity of beach sands

**Fig 2:**
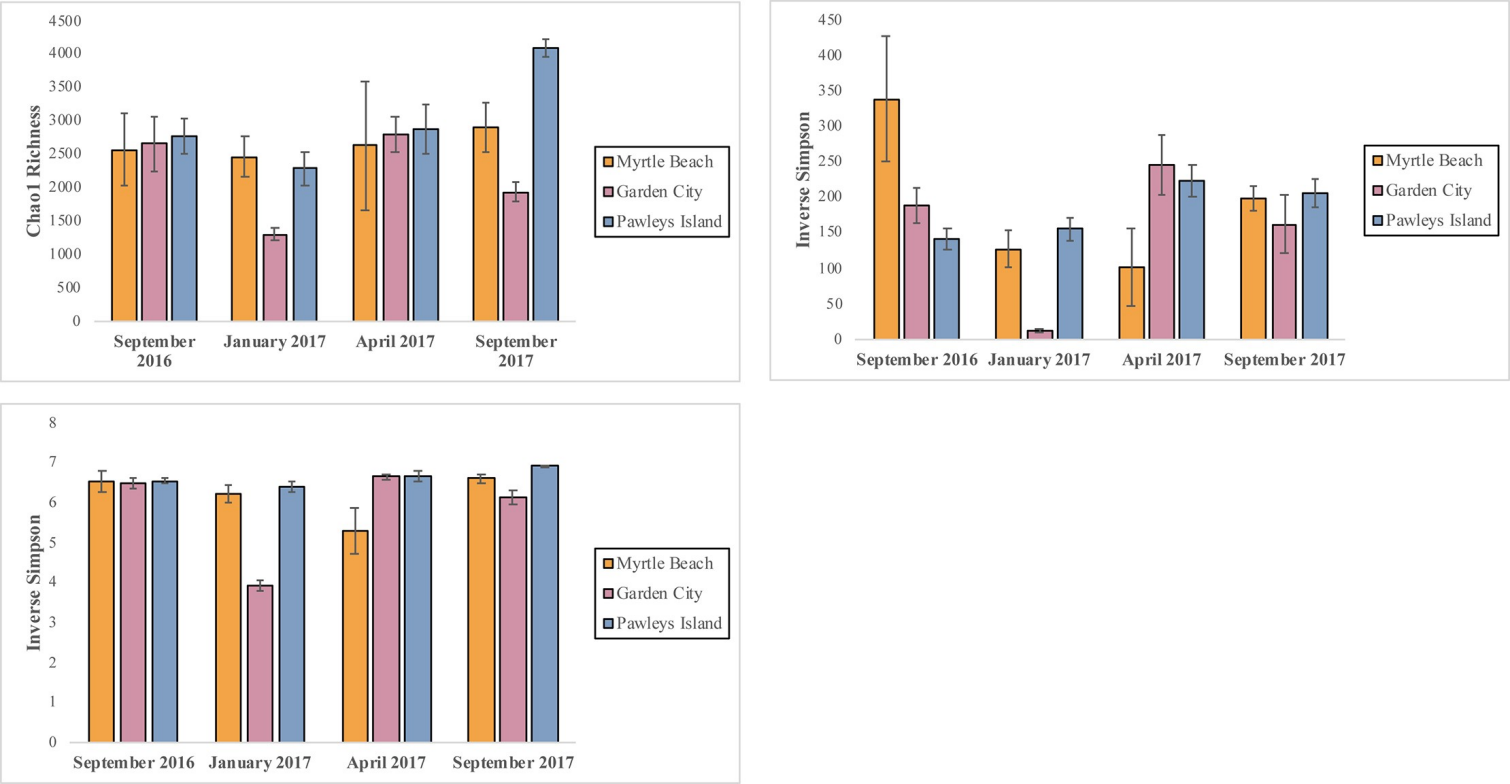
Alpha diversity metrics of microbial communities at each beach. Diversity plots showing the average Chao1 richness estimates (A), Inverse Simpson indices (B), and Shannon Diversity indices (C) for Myrtle Beach, Garden City, and Pawleys Island on each sampling date.

Richness and diversity measurements for each beach can been seen in Fig 2, which shows the estimated Chao1 richness (a), Inverse Simpson diversity index (b), and Shannon index (c) for each sample location during each sampling date. Myrtle Beach and Garden City communities showed the greatest disparity between richness and evenness compared to Pawleys Island, which saw comparable levels of diversity throughout the year. At Myrtle Beach, the community sampled on April 2017 was less diverse than on other sampling dates. Myrtle Beach additionally saw the greatest variation in richness and diversity between samples, as evaluated with standard error. At Garden City, the community sampled on January 2017 showed the lowest level of richness and diversity compared to the other sampling dates. Although diversity was mostly consistent temporally on Pawleys Island, the community sampled on September 2017 showed an overall higher richness than the other sampling dates. Other diversity indices and data from individual samples can be viewed in S4–S6 Tables. Statistical analysis of alpha diversity via QIIME2 found the community at Pawley’s Island to be significantly richer and more diverse via Shannon index (p < 0.01) compared to the other two beaches (S4 Fig), based on a Kruskal-Wallis pairwise comparison. Pawleys Island was not significantly different from MB in estimates of Faith’s Phylogenetic Diversity (PD), however. When grouped by season, the alpha diversity metrics (Richness, Shannon, and Faith’s PD) of Summer 2016 samples were significantly greater than Winter 2017 samples (p < 0.05). There were no other significant differences between the richness and diversity of grouped seasons. Statistical analysis via Spearman correlation revealed a small but significant (p < 0.05) correlation between water temperature and alpha diversity of samples.

**Fig 3:**
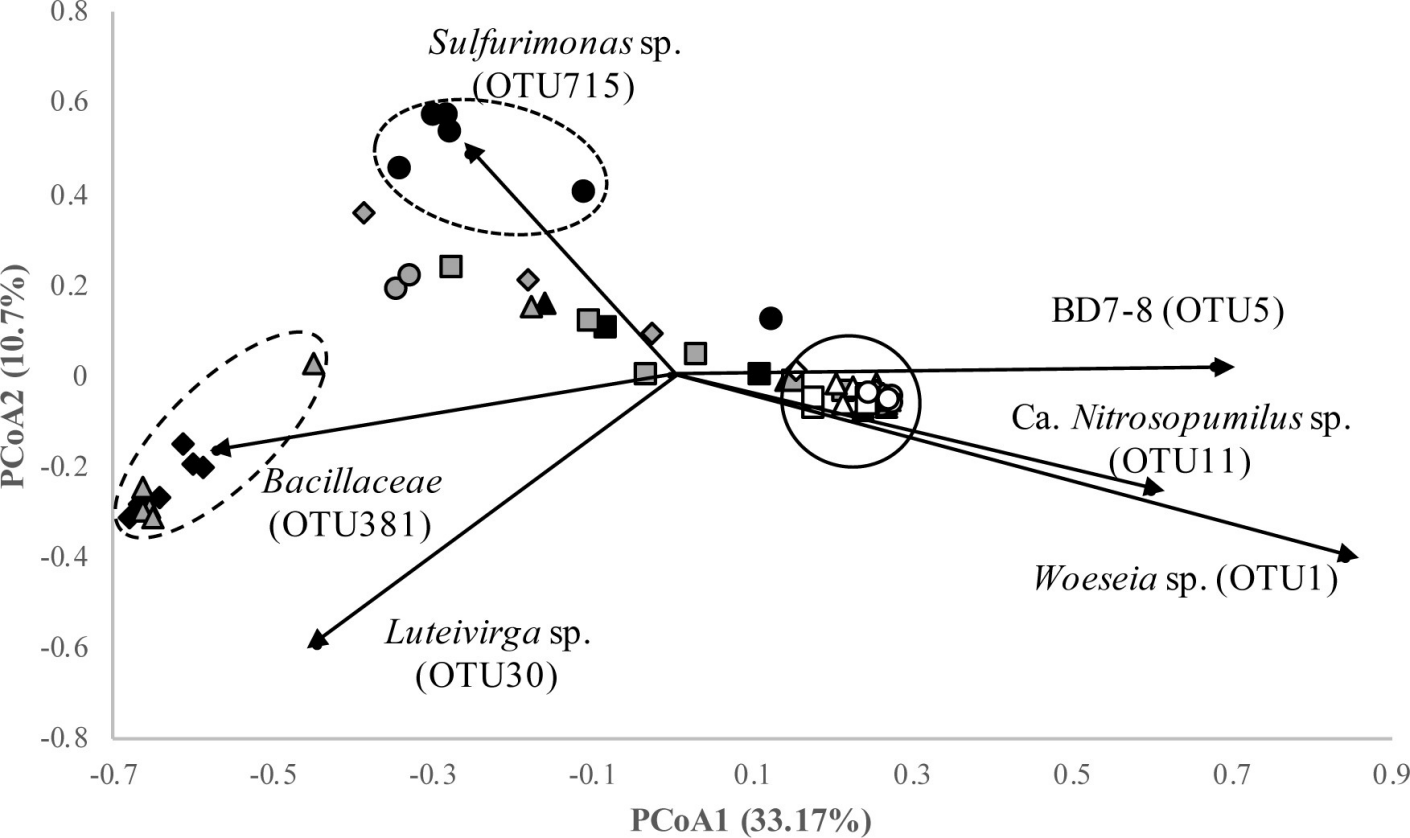
Principal Coordinate Analysis of microbial community structure from all samples based on a subsample of 7,676 sequences determined via theta-yc dissimilarity. Myrtle Beach samples are depicted as dark gray, Garden City as black, and Pawleys Island as white. Taxa displayed are those that significantly influenced (p < 0.001) the ordination of the different samples. Clustering was determined via Analysis of Molecular Variance (AMOVA) using mothur. Shapes correspond to particular sampling dates: squares for September 2016, diamonds for January 2017, triangles for April 2017, and circles for September 2017.

With regard to beta diversity, a pair-wise PERMANOVA pseudo-F test indicated significant differences (p < 0.01) in the community structure of Pawleys Island compared to the other two beaches, based on unweighted and weighted UniFrac analyses performed via mothur and QIIME2. No significant differences were observed in the community structures of Garden City and Myrtle Beach using the same analyses. When grouped according to season, significant differences in community structure were found between individual sampling dates except between Winter (January 2017) and Spring (April 2017). Principal Coordinate Analysis (PCoA) of all samples (Fig 3) allowed for the visualization of more of these differences in beta diversity between samples and beaches. Based on analysis of molecular variance (AMOVA), there exists three distinct clusters that separate themselves from all other samples (p<0.05). Cluster 1 contains Garden City samples collected on September 2017, cluster 2 contains Garden City samples from January 2017 and Myrtle Beach samples from April 2017, and cluster 3 contains all of the Pawleys Island samples. This analysis revealed a higher level of stability in the community structure of Pawleys Island over time than the other two beaches. OTUs differentiating the structure of the Pawleys Island community from the other samples include BD7-8, *Woeseia* sp., and the thaumarchaeote *Candidatus* Nitrosopumilus sp. A *Sulfurimonas* sp. OTU is the primary driver in shaping the differences in community structure for cluster 1, and an OTU classified within the family *Bacillaceae* is the primary driver shaping the differences in cluster 2.

Samples from each beach were separated and reanalyzed using Principal Coordinate Analysis to determine beta-diversity differences between sampling dates on the same beach and to determine which OTUs were influencing the variation in the samples. These results are shown in S5 Fig. These analyses show similar patterns of clustering for samples of Myrtle Beach (S5A Fig) and Garden City (S5B Fig). However, when the other beaches were removed from the analysis, Pawleys Island showed distinct clustering based on sampling date (S5C Fig). The associated OTUs driving the distance between clustering can be viewed in these figures, as well.

### Influence of environmental parameters on diversity

**Fig 4:**
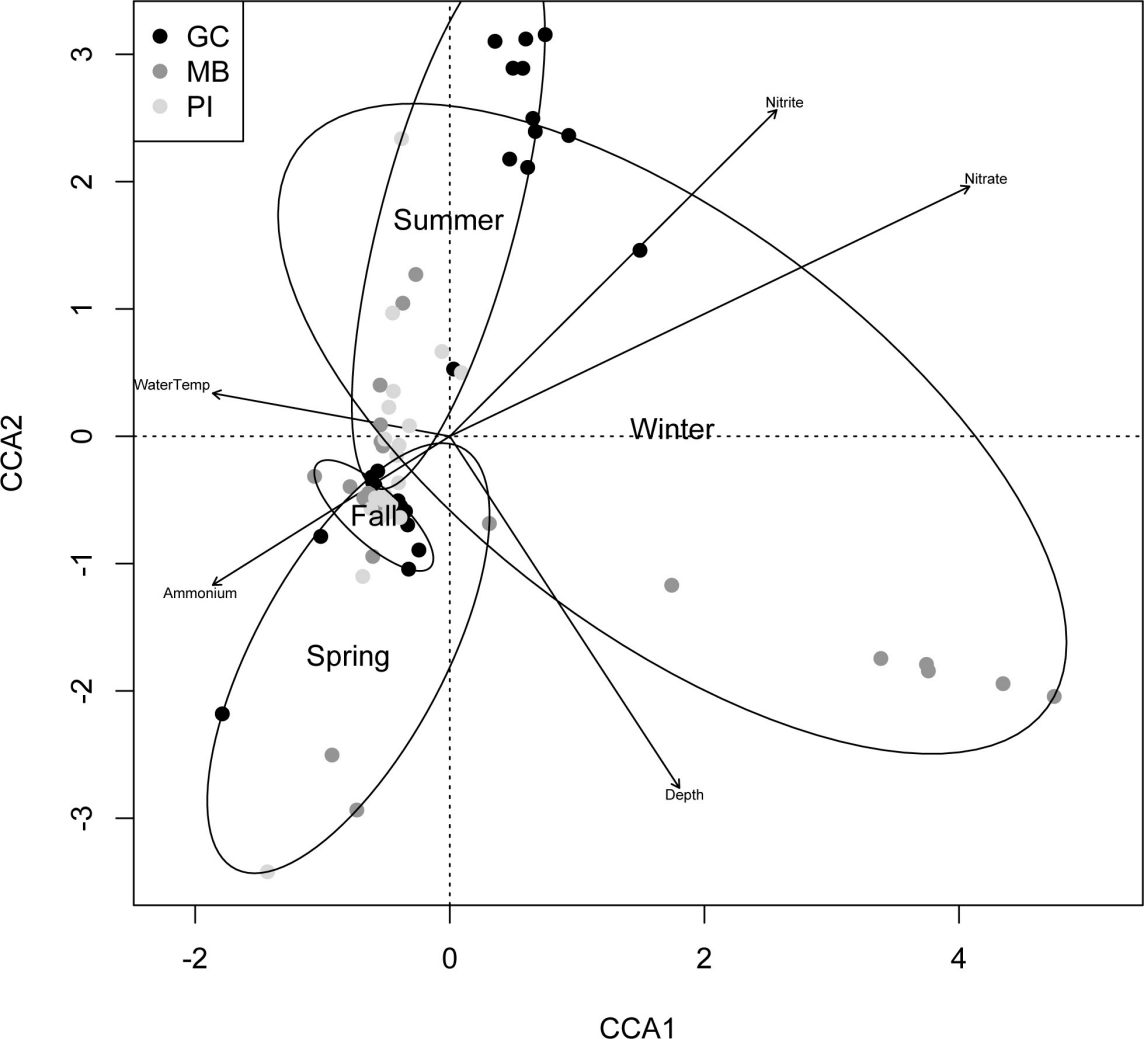
Canonical correspondence analysis (CCA) biplots of community structure of samples and environmental parameters. Parameters include water temperature, depth sampled, and concentrations of ammonium, nitrate, and nitrite in samples from Myrtle Beach (dark gray), Garden City (black), and Pawleys Island (light gray). Permutation tests were performed to determine significance of each parameter on community structure (p ≤ 0.05).

A canonical correspondence analysis (CCA) was performed to investigate the relationship between environmental parameters and the community structure of each sample (Fig 4). Samples were grouped according to sampling date based on which season they fell into according to the equinox calendar. Summer (September 2016), Spring (April 2017), and Fall (September 2017) produced significant clustering within the CCA plot, although the clustering of Spring samples was barely significant (p = 0.049). Pawleys Island samples again showed tighter clustering that those from the other two beaches. Water temperature, nitrate concentrations, and ammonium concentrations had the strongest influence (p<0.05) on the clustering of samples. Overall, environmental variables had the greatest influence on samples from Garden City (p = 0.002), and a much lower impact on samples from Myrtle Beach (p = 0.05) and Pawleys Island (p = 0.034).

## Discussion

These data on the structure of marine beach sand communities add to the knowledge base focused on microbial communities above the subtidal zone [13,14,24,26,29]. The bacteria and archaea detected in these three South Carolina beaches represent those of a subtropical recreational beach sand community subject to disturbances of varying degree by yearly tropical storms and add to the knowledge of beach sand communities of similar climates examined in Florida [14] and the Gulf Coast [24,29]. Examination of the community structure compared between all beaches and sampling dates revealed many regional similarities in both composition and diversity. However, this is not the case throughout the year, as we did see some differentiation in samples from Myrtle Beach and Garden City through comparison of community composition.

The most dominant bacterial phyla across this regional area is the *Proteobacteria*, particularly the *Gammaproteobacteria*, which has previously been found to comprise a majority of the communities within marine beach sands [12,13,14,24,26,29]. At least 85% of the bacterial community of each beach was comprised of members from the phyla *Proteobacteria*, *Planctomycetes*, *Acidobacteria*, *Firmicutes*, *Actinobacteria*, *Cyanobacteria*, *Bacteroidetes*, *Thaumarchaeota*, and *Chloroflexi*. Similarly, Halliday *et al*. [26] found *Acidobacteria*, *Actinobacteria*, *Bacteroidetes*, *Planctomycetes*, and *Proteobacteria* comprising over 90% of the bacterial community, while Boehm *et al*. [13] identified these phyla in addition to *Firmicutes* comprising approximately 90% of these communities. Similar to other beaches in the southeast of the United States [14,24,29], *Firmicutes* comprised a very small percentage (0.4%) of the bacterial community at Pawleys Island. Conversely, Myrtle Beach and Garden City saw significant though temporary increases in *Firmicutes* abundance on April 2017 and January 2017, respectively, comprising over 25% of the communities on these dates.

Common orders found at these beaches, included *Pirellulales*, *Steroidobacterales*, *Thermoanaerobaculales*, and *Actinomarinales*, which have been detected in other marine beach sand ecosystems across the United States [13,14,24,26]. Common families comprising these communities included *Pirellulaceae*, *Woeseiaceae* (formerly classified as *Sinobacteraceae*) [47], *Thermoanaerobaculaceae*, and *Anaerolinaceae*. This is similar to what has been found in the sands of other marine beaches [14,24,29], although variations in relative abundance of these taxa do exist based on the geographic location. For example, the *Planctomycetes* appeared to be more prevalent overall in South Carolina beach sands than those of other areas previously studied [14,24]. Many common genera recovered at these beaches have been established as common community members in marine beach sand ecosystems [19,24,26,29]. Conversely, *Blastopirellula* spp. and *Rubripirellula* spp. in particular were recovered abundantly in all South Carolina beach samples on all sampling dates, indicating that these may potentially be uniquely important regional members of these communities. Additionally, the abundance of *Thaumarchaeota* in these sands indicates the importance of nitrogen cycling, particularly aerobic ammonia oxidation, in these ecosystems [48].

Pawleys Island shows the most consistent diversity and richness over time, while Garden City showed the most variable diversity, with its lowest measured diversity following Hurricane Matthew. Communities at these beaches tended to have higher richness and diversity in summer compared to winter, which seems to correlate with increased activity of coastal picoplankton with temperature [49,50]. These data indicate that these ecosystems are highly diverse and at a level comparable to that of other marine beach sands in the southeastern region of the United States [14,24], on the lower end of the range found at several California beaches [13], and higher than what was detected on a Massachusetts beach [26]. Statistical analysis suggested that alpha diversity differed more according to beach location than sampling date, with Pawleys Island having a significantly higher level of richness and diversity than the other beaches. The consistency of alpha diversity estimates from this beach likely contributed to its differentiation from Myrtle Beach and Garden City. Additional sampling may be needed to establish if these patterns of diversity at these beaches are consistent over time and would alleviate this limitation in this study, as it is possible that the patterns in composition and diversity observed may be specific for this particular year of sampling.

Beta diversity analyses provided further evidence of the higher level of stability of the community at Pawleys Island and also revealed that compared to the other two beaches, the microbial community structure of Pawleys Island was distinct. Unlike alpha diversity, the beta diversity of these beaches was impacted by sampling date, with only the Winter (January 2017) and Spring (April 2017) samples not significantly differing from one another. These alpha and beta diversity analyses suggest that diversity and evenness are more dependent upon sampling location, while the overall community structure is dependent on both sampling date and location. The Garden City community appears the least stable of the three beaches, as it has the most differential clustering amongst the three beaches according to PCoA. The Garden City September 2017 community clusters separately from other samples, indicating that the renourishment project occurring at this beach was causing changes to the community structure, although it is unknown at this time how long lasting these effects will be. Samples from Garden City taken January 2017 and Myrtle Beach taken April 2017 cluster closely together and further away from all of the other samples, influenced heavily by OTUs of the family *Bacillaceae*, which seem to be contributing to the high degree of *Firmicutes* abundance in these samples. A BLAST search [37] classified these OTUs as an uncultured *Bacillus* sp. (99% identity) and *Bacillus algicola* (99% identity). *Bacillus algicola* was first discovered in association with the marine brown alga *Fucus evanescens* [51], and this OTU was prevalent in Garden City (>80,000 sequences recovered) in January 2017 and in Myrtle Beach (>10,000 sequences recovered) in April 2017. Thus, the increase in *Firmicutes* at these beaches suggests a potential introduction of these bacteria from debris brought by the Hurricane or may be a seasonal bloom that occurs on these two beaches. The presence of taxa associated with eukaryotes suggests a need to investigate the role of eukaryotic members of these communities like fungi or algae, which have been shown to be important members of these ecosystems [20].

The beach renourishment project at Garden City on September 2017 caused a distinct change in the community composition and structure. OTUs attributed to the sulfur-cycling bacteria *Thiomicrospira* (class *Gammaproteobacteria*) and *Desulfatiglans* (family *Desulfobacteraceae*) were found to differentiate these samples from the others at Garden City. These sulfur-cycling taxa are typically found in deep sea marine sediments [52,53], which is expected due to the source of the sand being added to the beach during this time period. Another taxon commonly found in deep sea marine habitats, *Sulfurimonas*, caused a shift in community structure differentiating these samples from those of other beaches [54]. This bacterium is widespread in marine systems and members of this genus are found to couple denitrification with sulfur oxidation [55]. These shifts due to sulfur cycling microbes indicates that sulfur cycling in beach sand ecosystems is distinctly different than that of deep-sea marine sediments and carried out by different taxa. These data additionally show that while beach renourishment projects attempt to restore the former structure of beaches to negate damage from erosion or hurricanes, they can impact the microbial communities in these sands, although it remains to be determined how permanent these changes are.

The compositional and diversity analyses suggest that the microbial community at Pawleys Island is highly stable and potentially more diverse comparatively, as the seasonal differences in community structure appear minimal compared to the other two beaches. These data show that regionally similar beaches may show distinct differences in communities, particularly when examined over time. This highlights the need for multi-date sampling in order to better compare two communities, as samples collected at the first time point (September 2016) likely would have discerned very minimal differences between the three beaches. The more secluded nature of Pawleys Island beach and its status as a true barrier island differentiate it from the other two beaches. These two factors may also contribute to the differences observed in this study compared to the other two beaches. The lower impact from anthropogenic factors and an increased physical isolation from other terrestrial ecosystems likely contributes to the community’s structure being more consistent and stable over time [56].

The measured environmental parameters appeared to have a small but significant (p < 0.05) effect on some of the most abundant OTUs among all samples, which agrees with previous work on diversity of sediment-based systems [57]. Environmental parameters had the greatest influence on communities from Garden City, likely because of the degree of change this ecosystem experienced throughout the year of sampling. The canonical correspondence analysis revealed that environmental parameters had the most significant influence on communities in September 2016 and September 2017 and did not significantly influence the community structure of January 2017 samples. These results reveal further patterns in seasonal community structure shifts with strong influences of sediment nitrogen concentrations. The significant positive correlation between alpha diversity estimates and water temperature lends further evidence to the existence of a seasonal fluctuation in community structure at these beaches.

This study expands our knowledge of microbial communities in United States marine beach sands above the subtidal zone [13,14,24,26], and is unique in that it analyzed the communities of three regionally similar beaches prior to and after a large-scale weather event that was particularly devastating to the area. From these data, it is apparent that marine beach sand communities in the Grand Strand area are compositionally diverse and that geographic and temporal factors may influence community structure and contribute to the establishment of microbial populations that are similar but unique to a particular beach within the same geographic region. The biggest changes in structure and diversity observed in these communities were driven by taxa not common across all sampling dates (*Firmicutes*). We have also shown differential stability in communities between beaches of the same geographic region, likely due to level of development, degree of tourism experienced, and the physical structures of the beaches themselves. Nevertheless, there were many similarities found in the composition of these communities and those of other beaches across the United States, including the high abundance of *Proteobacteria*, *Planctomycetes*, and nitrogen cycling taxa [12,13,14,19,24,26,29]. Further work is needed in order to understand how stable these bacterial and archaeal communities remain from year to year and establish what factors contribute to the stronger stability of certain communities compared to others, like that observed at Pawleys Island, as more samples taken across a longer duration of sampling would establish more patterns in composition and diversity of the three beaches and strengthen the conclusions regarding community similarity made herein. Expansion of the study to include microbial eukaryotes would provide more information on potential symbioses at play in these environments (competition, commensalism, etc.) that may influence changes in these communities.

## Supporting information

S1 Fig Microbial composition of taxa at the Phylum level for (a) Myrtle Beach, (b) Garden City, and (c) Pawleys Island on each sampling date. Individual samples are labelled based on the tidal zone (Supratidal, ST; High tide, HT; Mid-tide, MT; Low tide, LT) and relative sampling depth (cm) they were taken from.
(PDF)

S2 Fig Microbial composition of the ten most abundant taxa at the Order level for (a) Myrtle Beach, (b) Garden City, and (c) Pawleys Island on each sampling date. Individual samples are labelled based on the tidal zone (Supratidal, ST; High tide, HT; Mid-tide, MT; Low tide, LT) and relative sampling depth (cm) they were taken from.
(PDF)

S3 Fig Microbial composition of the ten most abundant taxa at the Family level for (a) Myrtle Beach, (b) Garden City, and (c) Pawleys Island on each sampling date. Individual samples are labelled based on the tidal zone (Supratidal, ST; High tide, HT; Mid-tide, MT; Low tide, LT) and relative sampling depth (cm) they were taken from.
(PDF)

S4 Fig Alpha diversity metrics of samples pooled for each beach, measured in number of observed OTUs (**a**), Shannon indices (**b**), and Faith’s Phylogenetic Diversity (**c**).
(PDF)

S5 Fig Principal Coordinate Analysis of bacterial diversity from all samples at collected from Myrtle Beach (**a**), Garden City (**b**), and Pawleys Island (**c**) based on a subsample of 6930 sequences per sample as determined via theta-yc dissimilarity. Taxa displayed are those that significantly influenced (p < 0.05) the ordination of the different samples. Clustering was determined via Analysis of Molecular Variance (AMOVA). Shapes correspond to particular sampling dates: squares for September 2016, diamonds for January 2017, triangles for April 2017, and circles for September 2017.
(PDF)

S1 Table Environmental parameters for sand samples from Myrtle Beach. ^a^ samples indicate location and relative depth (cm) from which samples were taken; ST = supratidal, HT = high tide, MT = mid-tide, LT = low tide. ^b^ values for temperature indicate the seawater temperature, therefore only one value is recorded in each column.
(PDF)

S2 Table Environmental parameters for sand samples from Garden City. ^a^ samples indicate location and relative depth (cm) from which samples were taken; ST = supratidal, HT = high tide, MT = mid-tide, LT = low tide^. b^ values for temperature indicate the seawater temperature, therefore only one value is recorded in each column.
(PDF)

S3 Table Environmental parameters for sand samples from Pawleys Island. ^a^ samples indicate location and relative depth (cm) from which samples were taken; ST = supratidal, HT = high tide, MT = mid-tide, LT = low tide. ^b^ values for temperature indicate the seawater temperature, therefore only one value is recorded in each column. ^c^ Data not available; unable to obtain samples for HT50 during the January 2017 sampling date.
(PDF)

S4 Table Alpha diversity of Myrtle Beach samples subsampled at 6930 sequences. (PDF)

S5 Table Alpha diversity of Garden City samples subsampled at 6930 sequences. (PDF)

S6 Table Alpha diversity of Pawleys Island samples subsampled at 6930 sequences. ^a^ No data for HT50 on the January 2017 sampling date.
(PDF)

S7 Table Ten most abundant OTUs at each beach and their closest BLAST match. (PDF)
