# Supplementary figures and images for: Microbial community structure shows differing levels of temporal stability in intertidal beach sands of the grand strand region of South Carolina
Source: PLoS One. 2020 Feb 27;15(2):e0229387. doi: 10.1371/journal.pone.0229387 (PMC7046189; doi:10.1371/journal.pone.0229387)

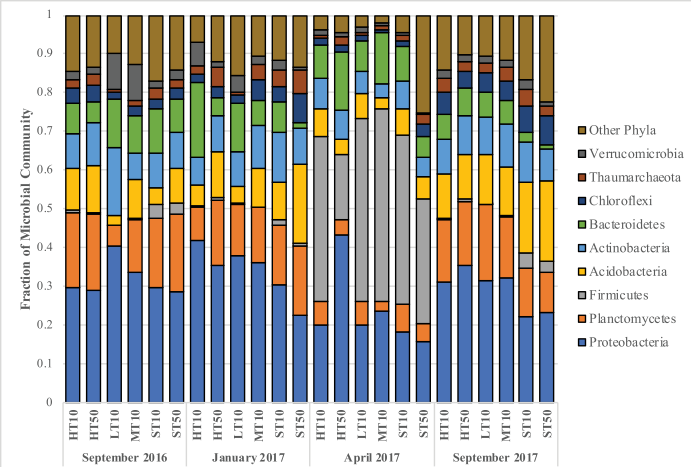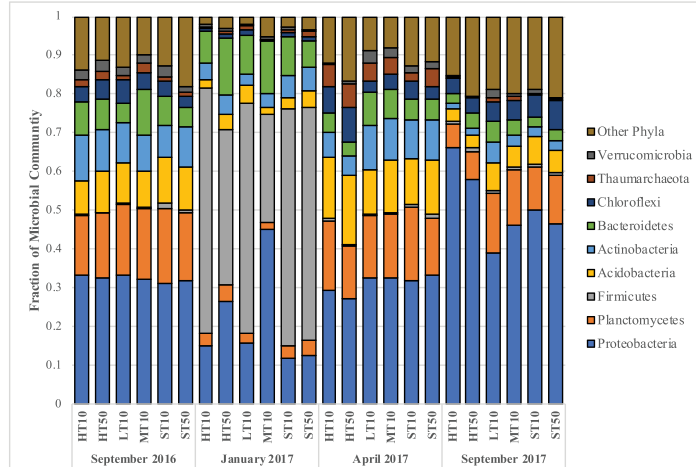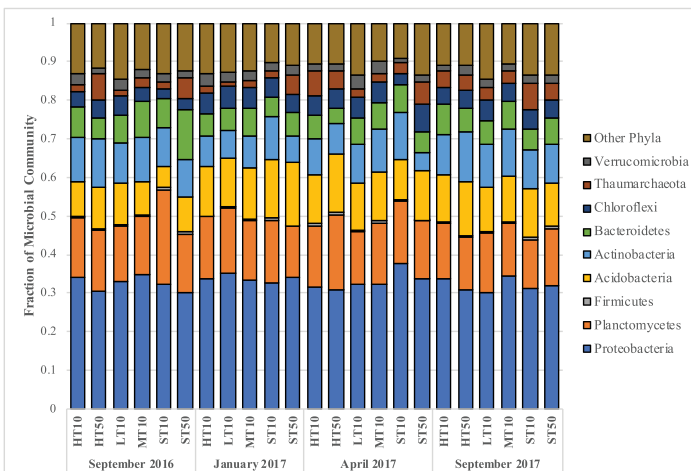

Supplement: S1 Fig — Microbial composition of taxa at the Phylum level for (a) Myrtle Beach, (b) Garden City, and (c) Pawleys Island on each sampling date. Individual samples are labelled based on the tidal zone (Supratidal, ST; High tide, HT; Mid-tide, MT; Low tide, LT) and relative sampling depth (cm) they were taken from. (PDF) [file pone.0229387.s001.pdf]

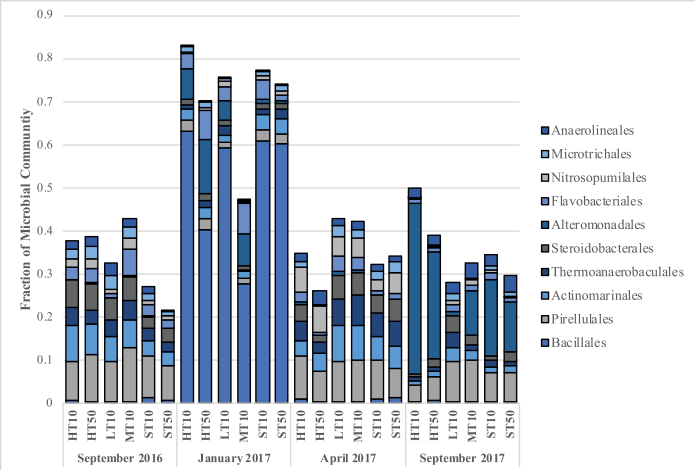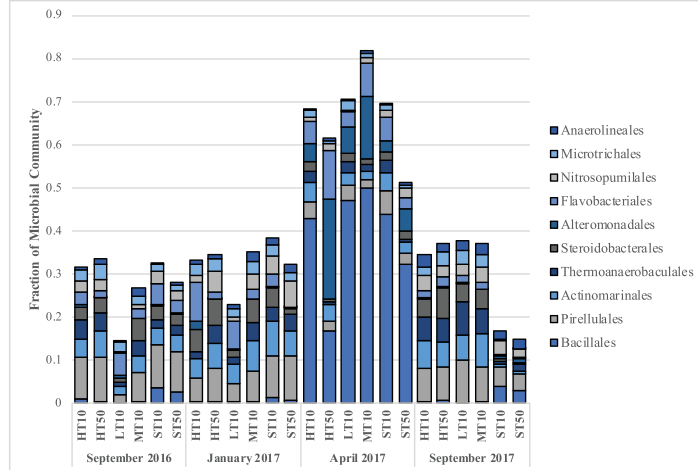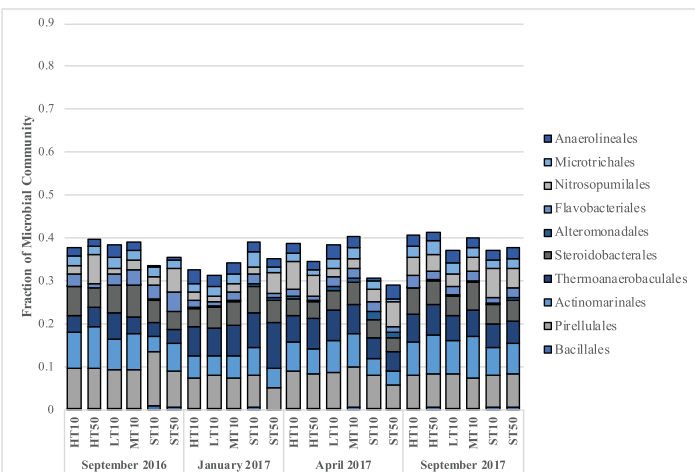

Supplement: S2 Fig — Microbial composition of the ten most abundant taxa at the Order level for (a) Myrtle Beach, (b) Garden City, and (c) Pawleys Island on each sampling date. Individual samples are labelled based on the tidal zone (Supratidal, ST; High tide, HT; Mid-tide, MT; Low tide, LT) and relative sampling depth (cm) they were taken from. (PDF) [file pone.0229387.s002.pdf]

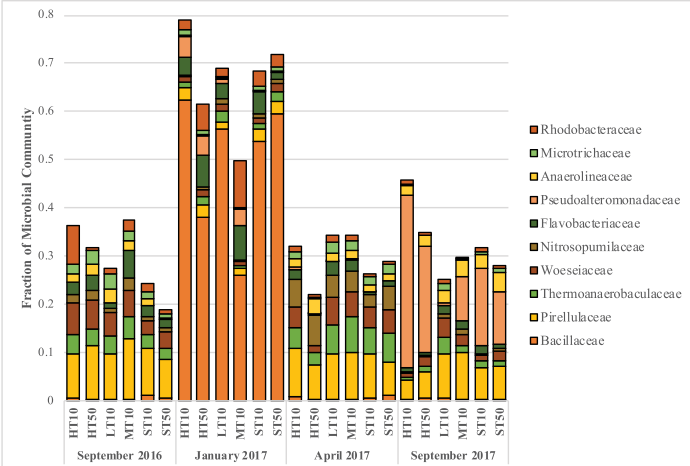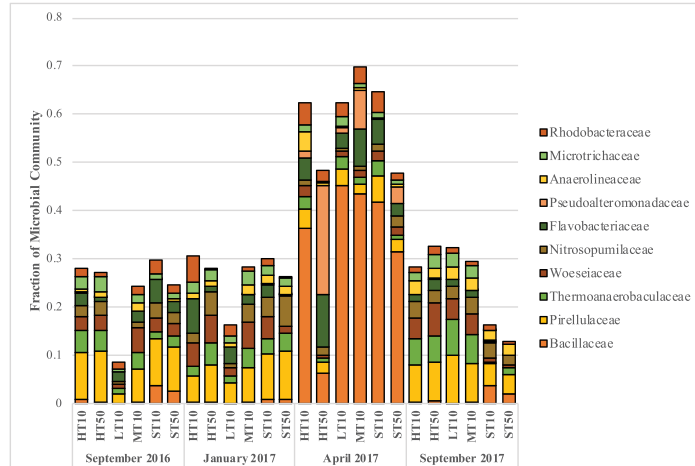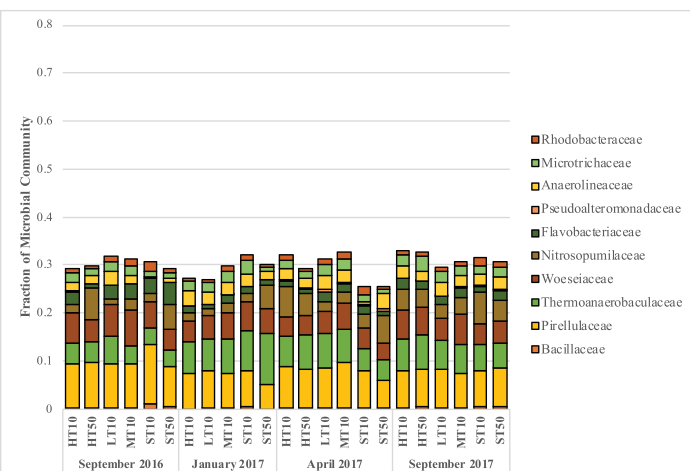

Supplement: S3 Fig — Microbial composition of the ten most abundant taxa at the Family level for (a) Myrtle Beach, (b) Garden City, and (c) Pawleys Island on each sampling date. Individual samples are labelled based on the tidal zone (Supratidal, ST; High tide, HT; Mid-tide, MT; Low tide, LT) and relative sampling depth (cm) they were taken from. (PDF) [file pone.0229387.s003.pdf]

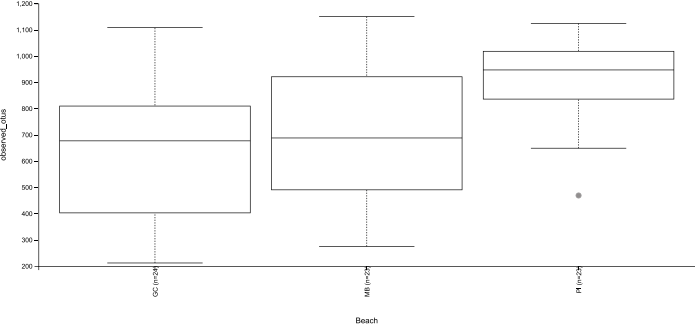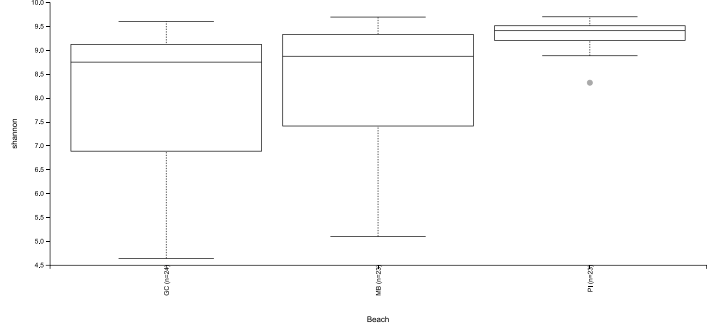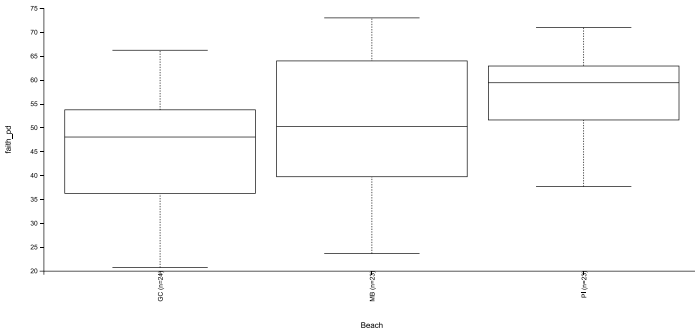

Supplement: S4 Fig — Alpha diversity metrics of samples pooled for each beach, measured in number of observed OTUs (a), Shannon indices (b), and Faith’s Phylogenetic Diversity (c). (PDF) [file pone.0229387.s004.pdf]

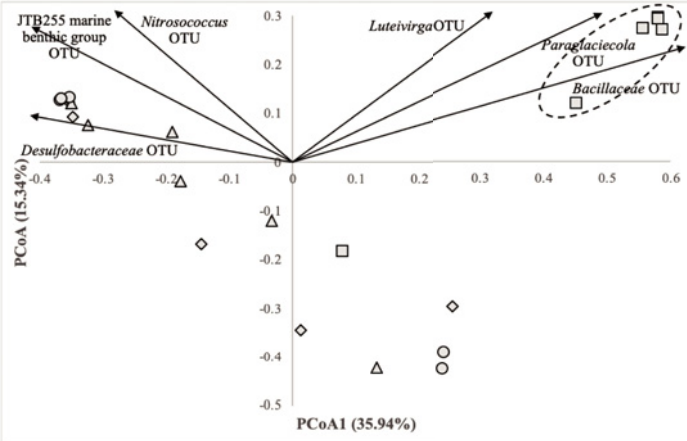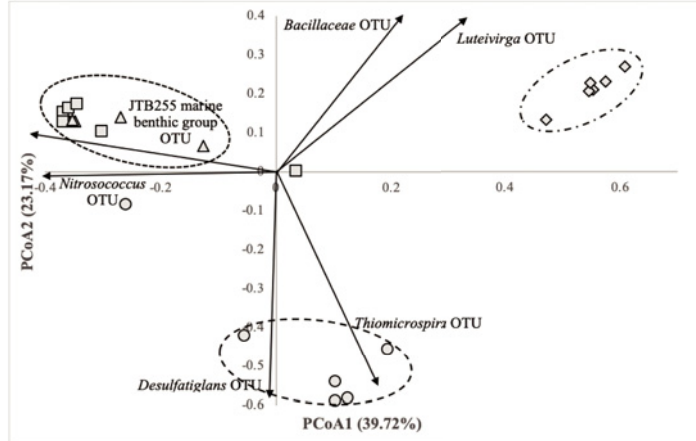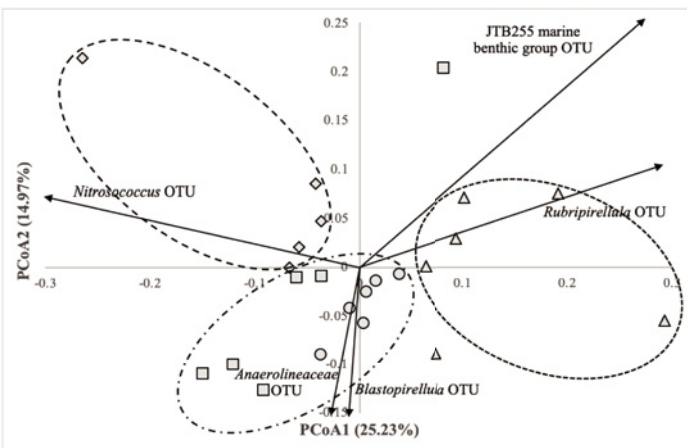

Supplement: S5 Fig — Principal Coordinate Analysis of bacterial diversity from all samples at collected from Myrtle Beach (a), Garden City (b), and Pawleys Island (c) based on a subsample of 6930 sequences per sample as determined via theta-yc dissimilarity. Taxa displayed are those that significantly influenced (p < 0.05) the ordination of the different samples. Clustering was determined via Analysis of Molecular Variance (AMOVA). Shapes correspond to particular sampling dates: squares for September 2016, diamonds for January 2017, triangles for April 2017, and circles for September 2017. (PDF) [file pone.0229387.s005.pdf]
