# Supplementary material for: Microbial community structure shows differing levels of temporal stability in intertidal beach sands of the grand strand region of South Carolina
Source: PLoS One. 2020 Feb 27;15(2):e0229387. doi: 10.1371/journal.pone.0229387 (PMC7046189; doi:10.1371/journal.pone.0229387)
Supplement: S3 Table — a samples indicate location and relative depth (cm) from which samples were taken; ST = supratidal, HT = high tide, MT = mid-tide, LT = low tide. b values for temperature indicate the seawater temperature, therefore only one value is recorded in each column. c Data not available; unable to obtain samples for HT50 during the January 2017 sampling date. (PDF) [file pone.0229387.s008.pdf]

Sample[a]	Seawater Temperature (°C)[b]				Sand Moisture Content (%)				Ammonium (nmol g^{-1} sand)				Nitrite (nmol g^{-1} sand)				Nitrate (nmol g^{-1} sand)			
	Sept. 2016	Jan. 2017	April 2017	Sept. 2017	Sept. 2016	Jan. 2017	April 2017	Sept. 2017	Sept. 2016	Jan. 2017	April 2017	Sept. 2017	Sept. 2016	Jan. 2017	April 2017	Sept. 2017	Sept. 2016	Jan. 2017	April 2017	Sept. 2017
ST10	28	13.4	22.6	27.2	5.06	18.46	5.11	4.83	31.42	47.53	29.51	29.51	3.13	0.86	1.85	2.21	268	248.4	131.5	240.3
ST50					8.47	18.13	24.32	8.83	47.53	36.79	145.2	79.77	3.18	0.54	0.17	5.1	251.7	228.8	278.5	137.4
HT10					5.32	19.39	6.92	7.74	40.37	49.32	39.98	26.37	2.56	0.62	0.63	1.83	281.1	238.6	131.5	125.6
HT50[c]					11.99		18.02	12.35	36.8		31.6	60.92	2.42		0.67	3.07	228.8		143.3	122.7
MT10					11.89	19.48	18.24	16.49	27.85	40.37	27.42	76.63	1.48	0.47	0.24	3.17	294.2	248.4	131.5	128.5
LT10					11.34	18.46	21.30	19.30	29.63	45.74	56.73	26.37	1.44	0.82	1.06	1.81	310.5	258.2	131.5	131.5
