# Supplementary material for: Microbial community structure shows differing levels of temporal stability in intertidal beach sands of the grand strand region of South Carolina
Source: PLoS One. 2020 Feb 27;15(2):e0229387. doi: 10.1371/journal.pone.0229387 (PMC7046189; doi:10.1371/journal.pone.0229387)
Supplement: S4 Table — (PDF) [file pone.0229387.s009.pdf]

			Mean Richness Index ± SD		Mean Diversity Index ± SD	
Sampling Date	Sample	Richness	Chao1	ACE	Inverse Simpson	Shannon
September 2016	ST10	1059	1353 ± 87	1328 ± 62	201.38 ± 16.38	6.18 ± 0.03
	ST50	1119	1419 ± 86	1402 ± 63	260.89 ± 20.39	6.31 ± 0.03
	HT10	1512	2005 ± 112	2004 ± 88	266.89 ± 29.06	6.63 ± 0.03
	HT50	1405	1821 ± 99	1850 ± 83	211.7 ± 23.91	6.50 ± 0.03
	MT10	2620	4331 ± 244	4692 ± 245	631.97 ± 62.05	7.29 ± 0.03
	LT10	657	925 ± 98	888 ± 66	72.34 ± 4.89	5.33 ± 0.04
January 2017	ST10	2013	3136 ± 192	3277 ± 176	166.66 ± 21.72	6.77 ± 0.04
	ST50	1267	1888 ± 134	2024 ± 134	23.39 ± 1.56	5.13 ± 0.06
	HT10	1195	1652 ± 111	1682 ± 94	93.63 ± 6.95	5.89 ± 0.04
	HT50	1489	1927 ± 100	1958 ± 85	109.99 ± 13.42	6.44 ± 0.04
	MT10	1104	1343 ± 71	1344 ± 56	121.22 ± 12.86	6.14 ± 0.04
	LT10	1244	1622 ± 95	1628 ± 77	95.45 ± 8.08	6.02 ± 0.04
April 2017	ST10	712	898 ± 64	923 ± 57	18.47 ± 1.04	4.57 ± 0.05
	ST50	2622	5031 ± 332	7095 ± 344	124.44 ± 14.62	6.89 ± 0.05
	HT10	656	1029 ± 123	981 ± 84	16.07 ± 0.98	4.36 ± 0.05
	HT50	1618	2428 ± 164	2432 ± 130	264.63 ± 25	6.58 ± 0.03
	MT10	724	1072 ± 101	1155 ± 100	11.64 ± 0.66	3.97 ± 0.06
	LT10	616	883 ± 94	861 ± 67	9.95 ± 0.58	4.06 ± 0.06
September 2017	ST10	1411	2002 ± 189	2047 ± 111	234.66 ± 17.46	6.37 ± 0.03
	ST50	889	1116 ± 75	1103 ± 55	172.41 ± 11.83	5.94 ± 0.3
	HT10	1723	2485 ± 147	2592 ± 134	131.60 ± 15.62	6.53 ± 0.04
	HT50	1428	1879 ± 105	1877 ± 166	95.68 ± 11.15	6.33 ± 0.04
	MT10	2069	3559 ± 242	4735 ± 238	170.34 ± 20.31	6.71 ± 0.04
	LT10	1738	2502 ± 147	2609 ± 134	160.68 ± 18.67	6.58 ± 0.04
