# Supplementary material for: Microbial community structure shows differing levels of temporal stability in intertidal beach sands of the grand strand region of South Carolina
Source: PLoS One. 2020 Feb 27;15(2):e0229387. doi: 10.1371/journal.pone.0229387 (PMC7046189; doi:10.1371/journal.pone.0229387)
Supplement: S5 Table — (PDF) [file pone.0229387.s010.pdf]

Sampling Date	Sample	Richness	Mean Richness Index ± SD		Mean Diversity Index ± SD	
			Chao1	ACE	Inverse Simpson	Shannon
September 2016	ST10	1187	1680 ± 132	1588 ± 83	238.04 ± 22.33	6.33 ± 0.03
	ST50	757	1027 ± 94	978 ± 60	129.12 ± 6.90	5.60 ± 0.03
	HT10	2059	3641 ± 260	4777 ± 240	100.48 ± 11.40	6.55 ± 0.04
	HT50	1427	1910 ± 113	1896 ± 86	119.20 ± 13.21	6.34 ± 0.04
	MT10	1388	1841 ± 107	1847 ± 86	126.41 ± 13.27	6.30 ± 0.04
	LT10	1759	2495 ± 143	2574 ± 126	155.35 ± 18.48	6.60 ± 0.04
January 2017	ST10	783	1226 ± 120	1318 ± 120	12.93 ± 0.32	3.96 ± 0.06
	ST50	582	807 ± 80	812 ± 65	8.15 ± 0.38	3.64 ± 0.06
	HT10	561	813 ± 86	869 ± 83	7.73 ± 0.33	3.41 ± 0.06
	HT50	685	970 ± 89	1007 ± 81	18.51 ± 0.94	4.25 ± 0.05
	MT10	478	657 ± 71	680 ± 63	20.21 ± 1.12	4.16 ± 0.04
	LT10	413	515 ± 48	529 ± 42	6.53 ± 0.32	3.42 ± 0.05
April 2017	ST10	1584	2321 ± 155	2294 ± 117	161.13 ± 18.47	6.50 ± 0.04
	ST50	1287	1606 ± 82	1651 ± 73	135.67 ± 14.91	6.28 ± 0.04
	HT10	1059	1343 ± 86	1309 ± 59	176.41 ± 15.94	6.18 ± 0.03
	HT50	1722	2377 ± 133	2392 ± 109	370.33 ± 28.12	6.72 ± 0.03
	MT10	1924	3043 ± 197	3149 ± 174	156.14 ± 18.13	6.62 ± 0.04
	LT10	1723	2512 ± 153	2578 ± 133	134.43 ± 14.39	6.47 ± 0.04
September 2017	ST10	698	773 ± 34	785 ± 30	94.92 ± 6.14	5.52 ± 0.3
	ST50	1117	1439 ± 89	1445 ± 71	118.06 ± 10.78	6.02 ± 0.04
	HT10	1132	1512 ± 95	1571 ± 88	21.55 ± 1.46	5.10 ± 0.06
	HT50	1323	1680 ± 87	1717 ± 76	96.52 ± 7.24	5.99 ± 0.04
	MT10	1065	1261 ± 63	1245 ± 45	188.75 ± 11.91	6.11 ± 0.03
	LT10	1364	1664 ± 79	1660 ± 61	220.33 ± 19.40	6.45 ± 0.03
