# Supplementary material for: Microbial community structure shows differing levels of temporal stability in intertidal beach sands of the grand strand region of South Carolina
Source: PLoS One. 2020 Feb 27;15(2):e0229387. doi: 10.1371/journal.pone.0229387 (PMC7046189; doi:10.1371/journal.pone.0229387)
Supplement: S6 Table — a No data for HT50 on the January 2017 sampling date. (PDF) [file pone.0229387.s011.pdf]

| Sampling Date | Sample | Richness | Mean Richness Index ± SD | | Mean Diversity Index ± SD | |
|---|---|---|---|---|---|---|
| | | | Chao1 | ACE | Inverse Simpson | Shannon |
| September 2016 | ST10 | 1185 | 1444 ± 74 | 1447 ± 58 | 139.45 ± 13.69 | 6.19 ± 0.03 |
| | ST50 | 1414 | 1794 ± 94 | 1790 ± 73 | 120.35 ± 11.04 | 6.29 ± 0.04 |
| | HT10 | 2008 | 3157 ± 200 | 3221 ± 168 | 102.05 ± 11.46 | 6.57 ± 0.04 |
| | HT50 | 1691 | 2208 ± 111 | 2211 ± 89 | 164.70 ± 18.63 | 6.58 ± 0.04 |
| | MT10 | 1866 | 2768 ± 164 | 2927 ± 155 | 86.54 ± 9.23 | 6.42 ± 0.04 |
| | LT10 | 1696 | 2336 ± 129 | 2412 ± 115 | 112.01 ± 12.15 | 6.43 ± 0.04 |
| January 2017[a] | ST10 | 1147 | 1388 ± 72 | 1386 ± 56 | 122.57 ± 12.19 | 6.17 ± 0.03 |
| | ST50 | 780 | 936 ± 58 | 948 ± 49 | 114.88 ± 8.61 | 5.74 ± 0.03 |
| | HT10 | 1540 | 2001 ± 106 | 1982 ± 81 | 148.52 ± 31.56 | 6.47 ± 0.04 |
| | MT10 | 1614 | 2219 ± 130 | 2206 ± 100 | 105.26 ± 21.21 | 6.39 ± 0.04 |
| | LT10 | 1479 | 1813 ± 82 | 1842 ± 70 | 139.71 ± 14.57 | 6.43 ± 0.04 |
| April 2017 | ST10 | 867 | 1071 ± 72 | 1043 ± 49 | 111.09 ± 9.85 | 5.85 ± 0.03 |
| | ST50 | 2007 | 2664 ± 125 | 2707 ± 106 | 238.32 ± 28.03 | 6.86 ± 0.03 |
| | HT10 | 1776 | 2495 ± 140 | 2566 ± 123 | 159.92 ± 16.40 | 6.55 ± 0.04 |
| | HT50 | 1320 | 1650 ± 87 | 1636 ± 66 | 207.12 ± 18.97 | 6.39 ± 0.03 |
| | MT10 | 1800 | 2495 ± 137 | 2556 ± 117 | 155.45 ± 17.21 | 6.61 ± 0.04 |
| | LT10 | 2131 | 3370 ± 206 | 3541 ± 189 | 217.59 ± 22.64 | 6.77 ± 0.04 |
| September 2017 | ST10 | 2273 | 3572 ± 211 | 3665 ± 182 | 219.26 ± 25.94 | 6.91 ± 0.04 |
| | ST50 | 2287 | 3816 ± 240 | 4853 ± 224 | 161.99 ± 18.46 | 6.79 ± 0.04 |
| | HT10 | 2073 | 3014 ± 161 | 3204 ± 157 | 138.56 ± 15.66 | 6.68 ± 0.04 |
| | HT50 | 2131 | 3198 ± 180 | 3341 ± 165 | 166.24 ± 18.64 | 6.74 ± 0.04 |
| | MT10 | 2246 | 3798 ± 245 | 4991 ± 232 | 132.65 ± 15.35 | 6.72 ± 0.04 |
| | LT10 | 2151 | 3372 ± 202 | 3559 ± 187 | 158.13 ± 18.02 | 6.73 ± 0.04 |
