# Supplementary material for: Microbial community structure shows differing levels of temporal stability in intertidal beach sands of the grand strand region of South Carolina
Source: PLoS One. 2020 Feb 27;15(2):e0229387. doi: 10.1371/journal.pone.0229387 (PMC7046189; doi:10.1371/journal.pone.0229387)
Supplement: S7 Table — (PDF) [file pone.0229387.s012.pdf]

Myrtle Beach								
OTU #	Sequences	Kingdom	Phylum	Order	Family	Genus	BLAST Match	% ID
3	36676	Bacteria	Firmicutes	Bacillales	Bacillaceae	Bacillaceae_unclassified	*Bacillus* sp.	98.4
1	36153	Bacteria	Proteobacteria	Steroidobacterales	Woeseiaceae	Woeseia	Uncultured gammaproteo	99.6
4	14508	Bacteria	Firmicutes	Bacillales	Bacillales_unclassified	Bacillales_unclassified	*Bacillus alcalophilus*	98.8
18	11739	Bacteria	Proteobacteria	SAR11_clade	Clade_I	Clade_Ia(98)	Uncultured SAR11 cluster	99.2
2	11708	Bacteria	Firmicutes	Bacillales	Bacillaceae	Bacillaceae_unclassified	*Bacillus algicola*	99.2
5	10387	Bacteria	Proteobacteria	BD7-8	BD7-8_fa	BD7-8_ge	Uncultured gammaproteo	98.8
10	9400	Archaea	Thaumarchaeota	Nitrosopumilales	Nitrosopumilaceae	Nitrosopumilaceae_ge	Uncultured marine archaeon	98.4
14	8903	Bacteria	Firmicutes	Bacillales	Planococcaceae	Planococcus	*Planococcus donghaensis*	98.8
9	8361	Bacteria	Proteobacteria	Alteromonadales	Pseudoalteromonadaceae	Pseudoalteromonas	*Pseudoalteromonas carrageen*	99.2
11	7696	Archaea	Thaumarchaeota	Nitrosopumilales	Nitrosopumilaceae	Candidatus_Nitrosopumilus	*Nitrosopumilus piranensis*	99.2
Garden City								
OTU #	Sequences	Kingdom	Phylum	Order	Family	Genus	BLAST Match	% ID
2	87028	Bacteria	Firmicutes	Bacillales	Bacillaceae	Bacillaceae_unclassified	*Bacillus algicola*	99.2
3	57864	Bacteria	Firmicutes	Bacillales	Bacillaceae	Bacillaceae_unclassified	*Bacillus* sp.	98.4
4	51173	Bacteria	Firmicutes	Bacillales	Bacillales_unclassified	Bacillales_unclassified	*Bacillus alcalophilus*	98.8
1	32456	Bacteria	Proteobacteria	Steroidobacterales	Woeseiaceae	Woeseia	Uncultured gammaproteo	99.6
8	17811	Bacteria	Firmicutes	Bacillales	Bacillales_unclassified	Bacillales_unclassified	Uncultured Firmicutes	98.8
13	17745	Bacteria	Proteobacteria	Alteromonadales	Pseudoalteromonadaceae	Psychrosphaera	*Psychrosphaera* sp.	98.4
6	14184	Bacteria	Proteobacteria	Rhodobacterales	Rhodobacteraceae	Rhodobacteraceae_uncl	*Loktanella* sp.	99.2
9	13412	Bacteria	Proteobacteria	Alteromonadales	Pseudoalteromonadaceae	Pseudoalteromonas	*P. carrageenovora*	99.2
28	10495	Bacteria	Proteobacteria	Alteromonadales	Pseudoalteromonadaceae	Psychrosphaera	*Psychrosphaera* sp.	96.8
21	9207	Bacteria	Proteobacteria	Alteromonadales	Alteromonadaceae	Alteromonas	*Alteromonas stellipolaris*	98.8
Pawleys Island								
OTU #	Sequences	Kingdom	Phylum	Order	Family	Genus	BLAST Match	% ID
1	62217	Bacteria	Proteobacteria	Steroidobacterales	Woeseiaceae	Woeseia	Uncultured gammaproteo	99.6
5	15943	Bacteria	Proteobacteria	BD7-8	BD7-8_fa	BD7-8_ge	Uncultured gammaproteo	98.8
7	13150	Bacteria	Acidobacteria	Thermoanaerobaculales	Thermoanaerobaculaceae	Subgroup_10	Uncultured Acidobacteria	98.4
11	8046	Archaea	Thaumarchaeota	Nitrosopumilales	Nitrosopumilaceae	Candidatus_Nitrosopumilus	*Nitrosopumilus piranensis*	99.2
10	7556	Archaea	Thaumarchaeota	Nitrosopumilales	Nitrosopumilaceae	Nitrosopumilaceae_ge	Uncultured marine archaeon	98.4
12	7500	Bacteria	Actinobacteria	Actinomarinales	uncultured	uncultured_ge	Uncultured actinobacterium	98.4
17	7104	Bacteria	Proteobacteria	Myxococcales	Sandaracinaceae	uncultured	*Sandaracinus* sp.	99.2
15	6486	Bacteria	Planctomycetes	Pirellulales	Pirellulaceae	Rubripirellula	Uncultured planctomycete	98.8
26	5952	Bacteria	Bacteroidetes	Flavobacteriales	Flavobacteriaceae	Muriicola	*Muriicola* sp.	97.2
22	5750	Archaea	Thaumarchaeota	Nitrosopumilales	Nitrosopumilaceae	Nitrosopumilaceae_uncl	Uncultured archaeon	98.4
